# Intracranial Venous Hypertension and Venous Sinus Stenting in the Modern Management of Idiopathic Intracranial Hypertension

**DOI:** 10.3390/life11060508

**Published:** 2021-05-31

**Authors:** Robert K. Townsend, Kyle M. Fargen

**Affiliations:** Department of Neurosurgery, Wake Forest University, Winston-Salem, NC 27101, USA; kfargen@wakehealth.edu

**Keywords:** venous manometry, venous sinus stenting, pseudotumor cerebri, idiopathic intracranial hypertension

## Abstract

Idiopathic intracranial hypertension (IIH) is a debilitating condition that has traditionally been difficult to treat. In recent years, there has been increasing focus on the role of intracranial venous hypertension in the pathophysiology of IIH. Based on increased understanding of this pathophysiology, venous sinus stenting (VSS) has emerged as a safe and reliable treatment for a certain population of patients with IIH. Stratifying patients with IIH based on the status of their venous outflow can provide insight into which patients may enjoy reduction in their symptoms after VSS and provides information regarding why some patients may have symptom recurrence. The traditional view of IIH as a disease due to obesity in young women has been cast into doubt as the understanding of the role of intracranial venous hypertension has improved.

## 1. Introduction

Idiopathic intracranial hypertension (IIH), previously referred to as pseudotumor cerebri, or more recently chronic intracranial venous hypertension syndrome (CIVHS) [1], is a condition characterized by a combination of intractable headaches, papilledema, visual symptoms, tinnitus, and elevated cerebrospinal fluid (CSF) opening pressure (OP) on lumbar puncture (LP) in the absence of an intracranial mass. This is a debilitating condition that has traditionally been managed relatively unsuccessfully with a variety of treatments, including high doses of carbonic anhydrase inhibitors, weight loss, CSF shunting procedures, and optic nerve sheath fenestration surgeries. In recent years, our understanding of intracranial venous physiology has led to recognition of intracranial venous hypertension as the pathophysiologic driver of IIH [1]. This has led to the development of venous sinus stenting (VSS) as a safe and effective treatment option for patients with IIH and a documented trans-stenosis pressure gradient (TSPG) [2].

While VSS was first reported as a successful treatment for IIH in 2002 [3], only recently have we begun to piece together the pathophysiology of intracranial venous hypertension and how it relates to the clinical syndrome of IIH. Part of the standard evaluation of IIH has come to include cerebral angiography with venous manometry [4], which has provided further insight into the pathophysiology of IIH and the different mechanisms through which individual patients develop high intracranial venous pressures. The pathophysiologic mechanisms leading to these elevated pressures continue to play a role after VSS, and our improved understanding of these mechanisms continues to influence post-operative management strategies.

The modern understanding of IIH as result of pathophysiologic impaired cranial venous drainage has its basis in a good understanding of intracranial pressure (ICP), dural venous outflow, and venous manometry. Recent calls for a new nomenclature system identifying IIH as CIVHS are also clarified with a deepened understanding of intracranial venous hypertension. In this manuscript, we aim to synthesize selected literature on these topics to provide a commentary on the modern evaluation and management of patients with IIH, the utility of VSS in their treatment, and areas of future research.

## 2. Discussion

### 2.1. Intracranial Venous Physiology and the Relationship to Intracranial Pressures

Intracranial CSF pressures and intracranial venous pressures are coupled by arachnoid granulations, which exist predominantly in the superior sagittal sinus (SSS). Animal studies have demonstrated that unidirectional flow of CSF from the subarachnoid space into the venous sinuses through these granulations occurs at a pressure gradient of 3–5 mmHg [5,6]. This understanding has informed the view that as intracranial venous pressure rises, the pressure within the subarachnoid space (ICP) will rise until it is 3–5 mmHg higher than the venous sinus, at which point CSF will drain across the arachnoid granulations. This equilibrium is the basis of the connection between intracranial pressure and venous sinus pressure.

While animal models have provided insight on the theoretical coupling of ICP and venous pressure, there is now robust literature demonstrating this relationship in human patients. One study of nearly 50 IIH patients correlating concomitant intracranial venous sinus pressures (in mmHg) with LP OP (in cm of water) in the lateral decubitus position [7] found that pressure measured at the torcula correlated in a one-to-one fashion with LP OP (r^2^ = 0.34, significant). While outliers were present (and are expected based on variability of arachnoid granulations, venous outflow anatomy, and potentially intracranial lymphatics), in general, OP and torcular pressures were consistently and reliably correlated. Importantly these values correlated across the OP spectrum regardless of whether the OP was in their teens or in their 50′s indicating that the correlation between OP and torcular pressures exists regardless of gender, body mass index (BMI), or presence/absence of venous sinus stenosis.

Further corroborating the relationship between venous sinus pressures and ICP are studies showing immediate reduction in ICP with procedures lowering venous pressures. This has been shown objectively in two separate studies in which patients who underwent simultaneous ICP monitoring and VSS demonstrated an immediate decrease in venous pressures and ICP after placement of the stent [8,9].

### 2.2. Normal Intracranial Venous Anatomy and Pressures

If patients with IIH have elevated OP secondary to high intracranial venous pressures, how do we define normal intracranial venous pressures? Remarkably, quite little is known about what normal venous sinus pressures should be; in fact, no studies exist measuring venous sinus pressures in humans in the absence of disease. We can, however, infer what we have learned by studying venous pressures in patients with symptoms of IIH with OP that are on the lower end of the OP spectrum.

Many patients presenting with symptoms of IIH now undergo venous manometry as part of the work-up to determine candidacy for VSS. Under light sedation, a guide catheter is usually advanced through the femoral vein into the internal jugular vein (IJ) and then a microcatheter is maneuvered into the SSS. Pressure is measured at the tip of the catheter through the fluid column within the microcatheter. The microcatheter is then withdrawn through the sinuses and pressures are measured at specific landmarks, terminating at the superior vena cava (SVC) to measure central venous pressure (CVP).

Studies of venous pressures in patients with IIH can provide some information on venous physiology and pressures in patients with normal OP. In one recent study, 104 patients presenting for evaluation of IIH underwent venous manometry [10]. Importantly, these patients were not screened with non-invasive imaging (MRV or CTV) and therefore represented an “all-comers” sample with IIH. Pressures and gradients between the SSS and torcula, torcula and transverse sinus (TS), TS and sigmoid sinus (SS), SS and IJ, and IJ to SVC (CVP) were described. Three critical principles were observed and described relating to venous physiology. First, CVP forms the foundation of intracranial venous pressures and, in the absence of vein narrowing, CVP is usually 4–5 mmHg lower than pressures measured in the SSS. This means that elevations in CVP, such as from obesity, will directly elevate intracranial venous pressures. Second, venous stenosis-related pressure gradients are very common in IIH and further elevate intracranial venous pressures beyond where they would be expected to be based on CVP. More than half (55%) of patients had transverse sinus pressure gradients of 8 mmHg further elevating intracranial venous pressures; over 80% of patients had a gradient of at least 4 mmHg across any adjacent anatomical site. Third, three patients had an OP of less than 20 and no adjacent pressure gradients, which simulates the non-diseased state. In these patients, SSS pressure was a mean of 16 mmHg and an overall pressure gradient of 4mmHg was present from SSS to CVP.

More recently, a study evaluating ICP and venous pressures in patients with IIH included 17 patients with OP of less than 20 cm of water [7]. In these patients, the mean SSS pressure was 13.5 mmHg. While limited, these data suggest that in the absence of transverse sinus stenosis or other focal area of venous stenosis, the pressure in the SSS should probably be 16–18 mmHg or less. A prospective study is currently underway studying venous sinus pressures in individuals without IIH but has not yet been completed.

### 2.3. IIH Is a Result of Elevated Intracranial Venous Pressures

As the understanding of the relationship between intracranial venous hypertension and ICP has deepened, it has become clear that IIH is not idiopathic at all [1]. We now know, from numerous studies measuring intracranial venous pressures in IIH patients, that elevated OP is consistently and uniformly commensurate with elevations in venous sinus pressures. In the study of 104 patients, all patients with OP greater than 20 had commensurate elevated venous pressures except for one patient with suspected IIH and OP of 30 that had previously had a craniotomy for pineal cyst and who was later diagnosed with chronic hydrocephalus given the dissociated OP and venous pressures [11]. The relationship between elevated OP and intracranial venous pressures in IIH is a fundamental observation that is consistently and repeatedly demonstrated with cerebral venography in IIH patients.

A new nomenclature and classification scheme has recently been proposed, calling for the name of the disease be changed to CIVHS [1] and to stratify patients based on the etiology of the venous hypertension. Under this new nomenclature, patients are stratified into four groups according to the drivers of venous hypertension: Elevations in venous sinus pressures can be entirely CVP-mediated, entirely venous stenosis-mediated, a combination of both, or related to post-thrombosis syndrome.

Central-type patients have elevated CVP without concomitant venous sinus stenosis, whereby morbid obesity or cardiorespiratory disease results in significant CVP elevations with subsequent elevations in intracranial venous pressures. These patients account for about 25% of patients with IIH, are not candidates for VSS, and tend to respond to weight loss or furosemide to reduce central venous volume and therefore CVP. Craniocervical-type patients demonstrate pathologic venous sinus stenosis with low-moderate CVP, wherein the venous outflow obstruction at the venous stenosis manifests as the primary driver of elevated intracranial venous pressures. These patients are often excellent candidates for VSS. Mixed-type patients are the largest subset of patients and demonstrate both pathologic venous sinus stenosis as well as moderate to high CVP, wherein both are independent and additive drivers of high intracranial venous pressures. Post-thrombotic patients demonstrate impaired intracranial venous outflow due to chronic venous sinus thrombosis and are the rarest.

Importantly, this nomenclature also describes “CIVHS spectrum disorder”, which is a condition wherein patients demonstrate LP OP of 15–24 cm of water but have serious symptoms of CIVHS that respond to pressure-lowering therapies, usually CSF diversion. This group accounts for about 15% of patients in the senior author’s practice. This group is not universally accepted among specialists and often have associated connective tissue disorders (most commonly Ehlers-Danlos syndrome) characterized by hyper-sensitivity to pressure. They are less commonly candidates for stenting as they often do not have a pathologic stenosis or TSPG, as would be predicted based on their lower OP and therefore lower venous sinus pressures.

### 2.4. Obesity and IIH

The longest-acknowledged pathophysiologic mechanism regarding patients with IIH has been obesity-related increased intra-abdominal pressure resulting in elevated CVP and upstream impairment of cranial venous outflow. As CVP forms the foundation in intracranial venous pressures, weight-related elevations in CVP will naturally cause higher intracranial venous pressures. However, CVP is rarely ever measured to be greater than 20 mmHg, even in the largest patients. In fact, it has been shown that CVP and BMI correlate in a linear fashion up to CVP of 20 mmHg [11]. As intracranial venous pressures are usually 4 or 5 mmHg higher than CVP in a normal situation, this means that even the most obese patients are unlikely to have venous sinus pressures greater than 25 mmHg in the absence of concomitant venous narrowing. Since venous sinus pressures are closely correlated to OP, mathematically, this practically means that most patients must have some other pathologic mechanism besides weight-related CVP elevation driving venous pressures higher if their OP is higher than 25 cm of water. It has been shown that an OP greater than 25 cm of water dramatically increases the chance that a significant venous sinus stenosis with TSPG of 8mmHg or more is discovered at time of venography [11].

### 2.5. Venous Sinus Stenosis Is a Result of a Positive Feedback Loop

The most common site of venous stenosis in patients with IIH is at the transverse sinus [1,12]. Transverse sinus stenosis has been observed in up to 93% of patients with IIH [13]. These patients usually have pulsatile tinnitus on the affected side, which becomes louder with worsened headache. There is ample evidence suggesting that transverse sinus stenosis is driven by a positive feedback loop in which an inciting event of elevation of ICP results in extramural compression of the dural venous sinus. The stenosis results in impaired venous outflow through the stenosed vein, resulting in venous congestion upstream of the stenosis with elevation of venous pressures within the upstream SSS, thereby resulting in further elevation in ICP. Increases in ICP cause further extramural compression of the transverse sinus, continuing the positive feedback cycle, which eventually leads to severe stenosis of the transverse sinus, high intracranial venous pressures upstream of the stenosis, and the clinical signs and symptoms of IIH [14]. This hypothesis is supported by a number of studies comparing pre-and post LP sinus calibers on invasive and non-invasive imaging. The most important and interesting case report used intravascular ultrasound and venous manometry to evaluate severe venous sinus stenosis in a patient with a very large TSPG. After measuring venous caliber and TSPG, a high-volume LP was performed and then the study was immediately repeated. Following LP, the TSPG and venous stenosis completely resolved, and intracranial venous pressures dropped dramatically [15].

Why does this positive feedback loop occur? The most reasonable hypothesis centers around several core principles: (1) There is an inherent predisposition that some people have to develop stenosis at the transverse sinus from extramural compression, which is probably related to the anatomy of the transverse sinus at that location; (2) elevations in ICP have a diffuse effect on venous sinus calibers, but the transverse sinus is most susceptible to becoming compressed; and (3) high ICP events must occur to precipitate this process. Most patients with IIH are overweight, and it is likely that nocturnal, episodic ICP elevations associated with obesity and sleep apnea instigate the positive feedback loop to occur in a recurrent manner, explaining why patients have only temporary relief following CSF removal.

Importantly, a small fraction of IIH patients are discovered to have venous outflow obstruction from intramural sinus stenosis. These patients often have large arachnoid granulations that have the appearance of round masses narrowing the sinuses. These granulations obstruct venous outflow, generate a TSPG, and can result in a similar clinical syndrome but behave differently than extramural compression from elevated ICP.

### 2.6. Treatment Considerations Based on High Venous Pressures

Treatments for IIH should now be considered based upon the way they influence ICP (and IIH symptoms) based on the described pathophysiologic mechanism. While carbonic anhydrase inhibitors continue to reduce ICP through reduction in CSF production, diuretics are effective through loss of volume and lowering of CVP, thereby reducing intracranial venous pressures. Weight loss similarly reduces CVP through lowering of intra-abdominal and intra-thoracic pressures, thereby lowering the foundation through which intracranial venous pressures arise. Furthermore, weight loss and correction of sleep apnea potentially reduce nocturnal ICP elevations that instigate venous sinus stenosis feedback loops.

CSF shunting procedures lower ICP through diversion of CSF out of the intracranial compartment and may mitigate dramatic elevations in venous sinus pressure by preventing the occurrence of venous sinus stenosis. However, once the shunt fails to drain appropriately, ICP increases, and symptoms return. VSS, on the other hand, is designed to disrupt the positive feedback loop by reinforcing the venous wall and preventing extramural compression. VSS therefore does not correct underlying elevations in CVP that are driving high venous pressures, but it corrects pathological stenoses that may result in dramatic elevations in OP.

### 2.7. Venous Sinus Stent Placement and Reduction of Intracranial Pressure

While guidelines for VSS candidacy are scarce, most practitioners offer VSS to patients with a TSPG of at least 8 mmHg [4]. Based on our knowledge of the relationship between OP and venous sinus pressures, resolution of a TSPG of 8 mmHg would amount to a commensurate reduction in OP of about 8 cm of water. After pre-loading dual antiplatelets, the patient is brought to the neurointerventional suite where they are put under general anesthesia. Angiography and venous manometry are performed to capture the baseline TSPG while under anesthesia (anesthesia is known to affect the venous pressures measured; therefore, venography is usually performed awake or under light sedation). Stents are then placed across the area of stenosis, and venous manometry is again performed to demonstrate resolution of the gradient and a reduction in upstream venous pressures.

The stent reinforces the walls of the transverse sinus and increases its resistance to extramural compression, restoring a more physiologic gradient of venous outflow. This helps lower ICP by preventing the development of upstream venous congestion. There are a few studies that quantified the reduction in CSF-OP with VSS. A study in 2019, which quantified this in 63 patients over 6 years, found that the average pre-treatment CSF-OP was with 37 cm of water, and post-treatment was 20.2 cm of water [16]. The average reduction was 16.8 cm of water, with the largest reduction of 50 cm of water. This study utilized a three-month pre-operative lumbar puncture and a three-month post-operative lumbar puncture as a marker for ICP, and these results have been corroborated in other similar studies as well [17]. In another study, similar results were seen with direct ICP monitoring during the VSS procedure [10].

### 2.8. Durability of Treatment and Quality of Life after Stenting

Multiple studies have sought to identify the rate of symptom recurrence and the rate of treatment failure after VSS. Meta-analyses, predominantly studying small retrospective series with short follow-up, suggest high rates of headache, tinnitus, and visual improvement. Nicholson et al. reported that nearly 80% of patients had improved headache, 90% had an improvement in tinnitus, and nearly 94% had improvement in papilledema [18]. The same study reported that symptomatic improvement was found to occur in 79.6% of patients, with a treatment failure rate (defined as need for repeat endovascular procedure or additional CSF diversion procedure) of only 12.4%. Other studies have reported similar rates [19,20].

A more realistic study was published in 2020 reporting on 81 patients that underwent VSS and used validated quality of life and headache scores [21]. Over half (54.3%) of patients either developed symptom recurrence after stenting or never felt better afterwards and underwent repeat LP after VSS to document change in OP from pre- to post-stenting. Even with recurrence of symptoms, however, quality of life and headache scores were significantly improved after VSS, and OP was, on average, 8–10 cm of water lower after stenting compared to pre-stenting values. Twenty-five percent underwent further surgical treatment for refractory symptoms. Only one patient underwent further surgical treatment for ongoing visual impairment, however, indicating that VSS appears to be a more durable treatment for IIH-related visual symptoms than for headache.

The recurrence of headache symptoms that occur after VSS is often in spite of the fact that OP is significantly lower than pre-stenting. We have labeled this observation the “re-equilibration” phenomenon [20]. Other patients may develop de novo stenosis outside of the stent or remotely in a new venous sinus, most commonly the SSS. A mechanism for new stenoses developing has been proposed [1]. This theory states that a new high ICP event occurs, driving extramural compression of an unreinforced portion of exposed sinus, this time in a different location (usually the medial transverse sinus or the S1 segment of the SSS closest to the torcula), again initiating a new positive feedback loop and resultant elevations in upstream venous pressures.

### 2.9. Other Procedures for IIH

The traditional understanding of IIH has focused on elevated intra-abdominal and intra-pleural pressures due to obesity. The increase in intracranial venous pressure and increase in CSF pressure results in headaches and papilledema, which can further lead to vision loss. Multiple treatment strategies have been employed to address these specific components of IIH, including bariatric surgery, CSF diversion via ventriculo-peritoneal shunting or lumbo-peritoneal shunting (VPS/LPS), and optic nerve sheath fenestration (ONSF). While there are no randomized trials to date to directly compare any of these procedures to each other or VSS, a recent systematic review provides some insight into the outcomes of these procedures [22]. ONSF is employed to address papilledema and visual deterioration specifically. CSF diversion procedures and VSS were demonstrated in this review to be very effective at relieving headache. Case reviews of patients undergoing bariatric surgery demonstrate significant relief in headaches and visual symptoms; however, this treatment works on the order of months and is thus inappropriate for patients with acutely deteriorating vision. While CSF diversion and ONSF were associated with high rates of treatment failure and/or inadequate initial symptom control, VSS was shown to be a relatively durable and effective treatment for HA, papilledema, and visual deterioration [22].

### 2.10. Future Directions

Future study is required in a number of areas. There are no cadaveric studies to demonstrate the hypothesized differential susceptibility of the TS to extramural compression in patients with IIH. A study to evaluate the relative elasticity or compliance in this population would provide much needed insight. A prospective FDA study evaluating a novel stent designed specifically for transverse sinus stenting (Serenity Medical, River stent) is underway. Historically, a number of medications (Vitamin A derivatives, antibiotics, steroids) have been linked to the development of IIH. How and why these medications affect venous pressures remain poorly understood. VSS is only one of a few surgical treatments available for patients with IIH. This list includes optic nerve sheath fenestration, CSF diversion via either VPS or LPS, and bariatric surgery. While systematic reviews have summarized the outcomes of these procedures [22], there are no randomized trials to date to compare treatment modalities and guide decision-making. A well-structured trial in this area would aid providers treating patients with IIH.

## 3. Conclusions

Research conducted over the past 20 years has provided new insight into the connection between intracranial venous pressure and ICP and how pathologic elevation of intracranial venous pressures results in the clinical syndrome of IIH. Thinking of IIH as a disease resulting from obesity in young women is antiquated and does not take into account venous drivers of the condition. VSS addresses one of the major pathophysiologic mechanisms of IIH, and it is worthwhile to include investigation of intracranial venous pressures to ensure that a potentially appropriate therapy is offered. While many physicians do consider VSS to be a possible first-line therapy for IIH, there still remain many unanswered questions regarding patient selection, surgical technique, and management of symptom recurrence. Future efforts will help answer these questions and provide insight into appropriate patient selection for the investigation and treatment of venous drivers of IIH.
